# Validation of a respiratory symptom score from the Quality of Life-Bronchiectasis Questionnaire in adults with *Mycobacterium avium* complex lung disease

**DOI:** 10.1080/1179271X.2026.2655469

**Published:** 2026-04-28

**Authors:** Daniel Serrano, Mariam Hassan, Marie-Laure Nevoret, Dayton W. Yuen, Shauna McManus, Lauren Podger, Sam Parsons, Bryant Barnes, Charles L. Daley, Kevin C. Mange

**Affiliations:** a The Psychometrics Team, London, UK; b Health Economics and Outcomes Research, Insmed Incorporated, Bridgewater, NJ, USA; c Clinical Development, Insmed Incorporated, Bridgewater, NJ, USA; d Patient Centered Outcomes, OPEN Health, Bethesda, MD, USA; e Patient Centered Outcomes, OPEN Health, London, UK; f Department of Medicine, National Jewish Health and the University of Colorado School of Medicine, Denver, CO, USA

**Keywords:** Respiratory symptoms, lung disease, *Mycobacterium avium* complex, patient-reported outcome, psychometric validation

## Abstract

**Objective:**

To assess the psychometric properties of a respiratory symptom score from the Quality of Life-Bronchiectasis Questionnaire in adults with *Mycobacterium avium* complex (MAC) lung disease.

**Methods:**

Structural validity, internal consistency, test-retest reliability, and known-groups validity were assessed using data from the ARISE (NCT04677543) and ENCORE (NCT04677569) phase 3 trials. Convergent validity was assessed using Exacerbations of Chronic Pulmonary Disease Tool (EXACT), EXACT Respiratory Symptoms (E-RS), St. George Respiratory Questionnaire (SGRQ), and Functional Assessment of Chronic Illness Therapy (FACIT) Fatigue Scale. Anchor-based meaningful within-patient change (MWPC) thresholds were estimated using the Patient Global Impression of Severity Respiratory scale.

**Results:**

In total, 362 patients were included for cross-sectional (ARISE: 97, ENCORE: 265) and longitudinal (ARISE: 99) validation analyses. Modern psychometric methods supported item relevance and an essentially unidimensional mean score. The respiratory symptom score demonstrated adequate internal consistency (McDonald’s omega: 0.86, Cronbach’s alpha: 0.81), test-retest reliability (intraclass correlation coefficient: 0.73), convergent validity (EXACT: −0.72, E-RS: −0.72, SGRQ: −0.77, FACIT-Fatigue: 0.51), and known-groups validity. The estimated MWPC threshold was a 16.67-point median change from baseline (95% CI: 8.33–16.83 points).

**Conclusions:**

The respiratory symptom score is a robust, sensitive, and responsive measure of respiratory symptoms in adults with a new or recurrent MAC lung disease diagnosis.

## Introduction

*Mycobacterium avium* complex (MAC) is the leading cause of nontuberculous mycobacteria (NTM) lung disease, an increasingly prevalent condition that often occurs in individuals with underlying lung conditions.^1–4^ Symptoms include cough, fatigue, chest pain, wheezing, shortness of breath, fever, weight loss, and hemoptysis, with pulmonary damage resulting in declining lung function.^1–4^ MAC lung disease is associated with substantial humanistic and economic burden.^5–7^

The inclusion of patient-reported outcome (PRO)-based endpoints in clinical trials is a key interest of patient-focused research and drug development in NTM lung disease, and patients have identified it as one of the top priorities for future research.^8–11^ However, there are no validated PRO instruments to evaluate respiratory symptoms in patients with MAC lung disease.^10^

The Quality of Life-Bronchiectasis (QOL-B) version 3.1 questionnaire has been validated in patients with bronchiectasis and gram-negative infection.^12^^,^^13^ It includes a 9-item respiratory domain (QOL-B RD) that assesses respiratory symptoms such as congestion, cough, wheezing, chest pain, and shortness of breath.^12^^,^^13^ Eight of these items assess the severity or frequency of respiratory symptoms over a 1-week recall period (e.g., from “a lot” to “not at all”), while one unique item (item 32 “sputum color”) assesses the appearance of sputum over a 1-week recall period using response options of different color gradations (e.g., clear, clear to yellow, brownish-dark).

Previously conducted qualitative concept elicitation research indicated that the respiratory symptoms that were most prevalent, bothersome, and important to patients with MAC lung disease are captured by the items in the QOL-B RD.^14^ Cognitive interview results confirmed the overall relevance, comprehensibility, and appropriateness of the QOL-B RD among patients with MAC lung disease; the recall period, response options, and concept attributes were suitable and meaningful to most patients.^14^

This psychometric validation endeavored to develop a fit-for-purpose endpoint supporting registrational efficacy determinations by the United States (US) Food and Drug Administration (FDA) within the MAC lung infection context of use. Because qualitative interviews demonstrated that the sputum color item had weaker endorsement, relevance, and meaningfulness compared to other items^14^ and sputum color is a clinical sign rather than a symptom exemplifying how a patient may feel or function, FDA’s division of clinical outcomes assessment (DCOA) recommended validating an 8-item respiratory symptom score (RSS). The 8-item RSS was identical to the QOL-B RD excepting the omission of the sputum color item (item 32). The prespecified psychometric validation analyses presented in this manuscript demonstrate evidence supporting the fit-for-purpose status of the 8-item respiratory symptom score in MAC lung disease. Some of the results of this study have been previously reported in the form of an abstract.^15^

## Methods

### Study design

**Figure 1.**
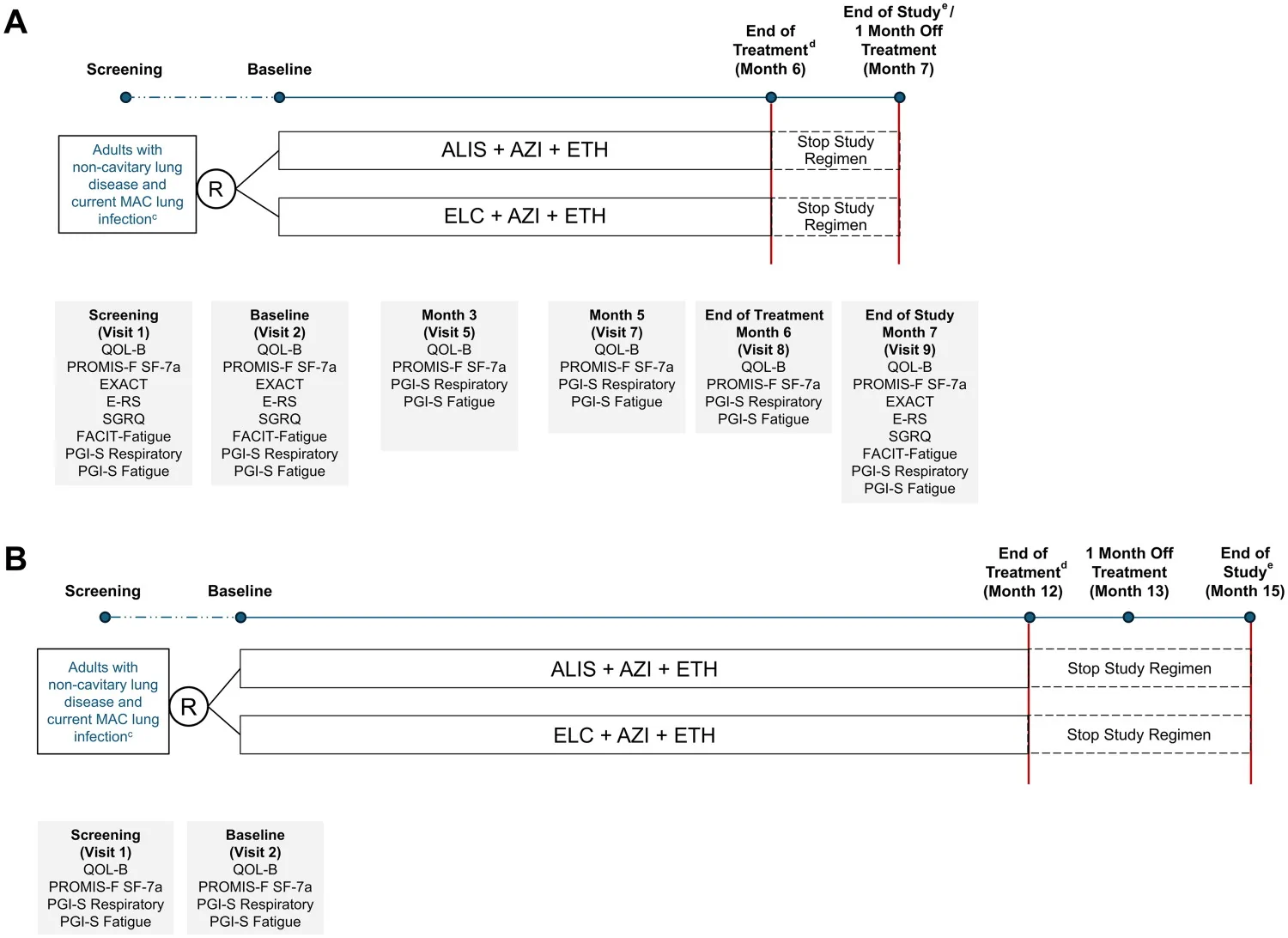
Study design and assessments^a^ for (A) ARISE and (B) ENCORE. ^a^End of study is defined as the date of the last study visit completed by a participant. End of treatment is defined as the date of the last dose of ALIS/ELC, AZI and/or ETH, whichever study drug dose is taken last. Abbreviations: ALIS, amikacin liposome inhalation suspension; AZI, azithromycin; ELC, empty liposome control; EOS, end of study; EOT, end of treatment; E-RS, Exacerbations of Chronic Pulmonary Disease Tool Respiratory Symptoms; ETH, ethambutol; EXACT, Exacerbations of Chronic Pulmonary Disease Tool; FACIT-Fatigue, Functional Assessment of Chronic Illness Therapy Fatigue Scale; MAC, *Mycobacterium avium* complex; PGI-S, Patient Global Impression of Severity; PRO, patient-reported outcome; PROMIS-F SF-7a, Patient-Reported Outcomes Measurement Information System Short Form v1.0 – Fatigue 7a; QOL-B, Quality of Life Bronchiectasis; R, randomization; SGRQ, St. George Respiratory Questionnaire.

Data were analyzed from the ARISE (NCT04677543) and ENCORE (NCT04677569) clinical trials (Figure 1).^16^^,^^17^ The study protocols were approved by Advarra Institutional Review Board (ARISE: protocol reference number Pro00045468, 12 August 2020; ENCORE: protocol reference number Pro00045569, 4 August 2020) and ethics approval was received from all study sites. All patients provided written informed consent. Details are available in the online Supplemental Material.

The entire QOL-B questionnaire was administered to all study participants, and the individual domains were scored according to the rights granted by the copyright holder. The 9-item QOL-B RD score is standardized on a scale from 0 to 100 points, with higher scores representing lower severity/frequency of symptoms.^13^ The 8-item respiratory symptom score is scored using the same methodology as the QOL-B RD and standardized on the same scale of 0 to 100 points. The 8 items included in the respiratory symptom score were severity of chest congestion, coughing during the day, and coughing up mucus, and frequency of shortness of breath with greater activity, wheezing, chest pain, shortness of breath when talking, and waking at night because of coughing. As noted above, the item assessing sputum color was omitted. Results of the psychometric validation of the 9-item QOL-B RD score are presented in the online Supplemental Material.

### PRO assessments

The Exacerbations of Chronic Pulmonary Disease Tool (EXACT),^18–20^ EXACT Respiratory Symptoms (E-RS),^21^^,^^22^ St. George Respiratory Questionnaire (SGRQ),^23^ and Functional Assessment of Chronic Illness Therapy (FACIT) Fatigue Scale^24^ were used as validators for the 8-item respiratory symptom score. The Patient Global Impression of Severity (PGI-S) Respiratory served as the primary anchor to analyze meaningful within-patient change (MWPC) in the respiratory symptom score. Additional information is provided in the online Supplemental Material.

### Statistical analysis

Psychometric analyses were conducted using R version 3.4.0 following a prespecified validation analysis plan developed by the first author independent of the sponsor. Full methodological details are provided in the online Supplemental Material.

#### Cross-sectional analyses

The cross-sectional validation included screening and baseline data from patients in ARISE and ENCORE who provided item-level QOL-B RD data at baseline. Analyses were conducted using baseline data from this full sample, except for convergent validity (baseline data from ARISE patients only) and test-retest reliability (screening to baseline data from ARISE patients only).

##### Item-level descriptive assessments

Item response patterns were evaluated for floor and ceiling effects (i.e. > 25% of participants endorsing the lowest/highest response categories) and response category sparseness (i.e. endorsed by < 10% of participants). Inter-item polychoric correlations were estimated to understand the pattern of respiratory symptom score item associations.

##### Modern psychometric methods

The domain structure was evaluated using item response theory (IRT) models. Model fit was evaluated using the C2-based $\chi^{2}$ and root mean squared error of approximation (RMSEA), Tucker-Lewis Index (TLI), and comparative fit index (CFI). RMSEA model fit criteria were based on classical tests of exact fit (lower confidence level [LCL] = 0) or close fit (LCL < 0.05),^25^ while TLI and CFI values ≥ 0.95 indicated acceptable model fit.^26^ Essential dimensionality and scoring statistics were employed to empirically determine the optimal scoring procedure for the respiratory symptom score (i.e. unit-weighted versus empirically-weighted and essentially unidimensional^27^ versus multidimensional) using McDonald’s omega ratios and the H statistic.^28^ A unit-weighted (e.g., sum score or mean score) essentially unidimensional^27^ score for the respiratory symptom score was supported if the omega ratio was ≥ 0.80 and H was ≥ 0.70.^29^ The presence of local dimensions and achievement of conditional independence of the items was evaluated using Chen and Thissen’s G^2^ statistic for local dependence across unidimensional, multidimensional, and bifactor structures.^30^ Item bias was evaluated using differential item functioning (DIF) and differential test functioning (DTF) statistics across reference and focal groups for age, sex, and MAC history, respectively: below vs. at or above median age, male vs. female sex, and initial vs. subsequent MAC lung infection.

##### Reliability and validity

Internal consistency was estimated using McDonald’s omega and Cronbach’s alpha (success criterion: ≥ 0.7), augmented with corrected item-to-total polyserial correlations (success criterion: ≥ 0.4).^31–33^ Test-retest reliability was estimated in a stable subset of patients reporting no change in PGI-S Respiratory between screening and baseline (up to 70-day retest interval) using the two-way random effects intraclass correlation coefficient (ICC[2,1]; success criterion: ≥ 0.7).^31^^,^^34^ Convergent validity was assessed using Pearson correlations (success criterion: |r| ≥ 0.4) between the respiratory symptom score and EXACT, E-RS, SGRQ, and FACIT-Fatigue at baseline.^31^ Known-groups validity was estimated by fitting a linear model to the baseline respiratory symptom score and comparing least squares means for the PGI-S Respiratory “low severity” group (reference) against each other PGI-S Respiratory severity group (effect) in pairwise contrasts. Acceptable known-groups validity was achieved if a preponderance of the known-effect-groups had ordered means and corresponding effect sizes > 5%.

#### Longitudinal analyses

The longitudinal validation analysis sample comprised patients enrolled in ARISE. QOL-B RD raw scores were imputed for patients in ARISE missing item-level data (see online Supplemental Material).

##### Meaningful change

**Figure 2.**
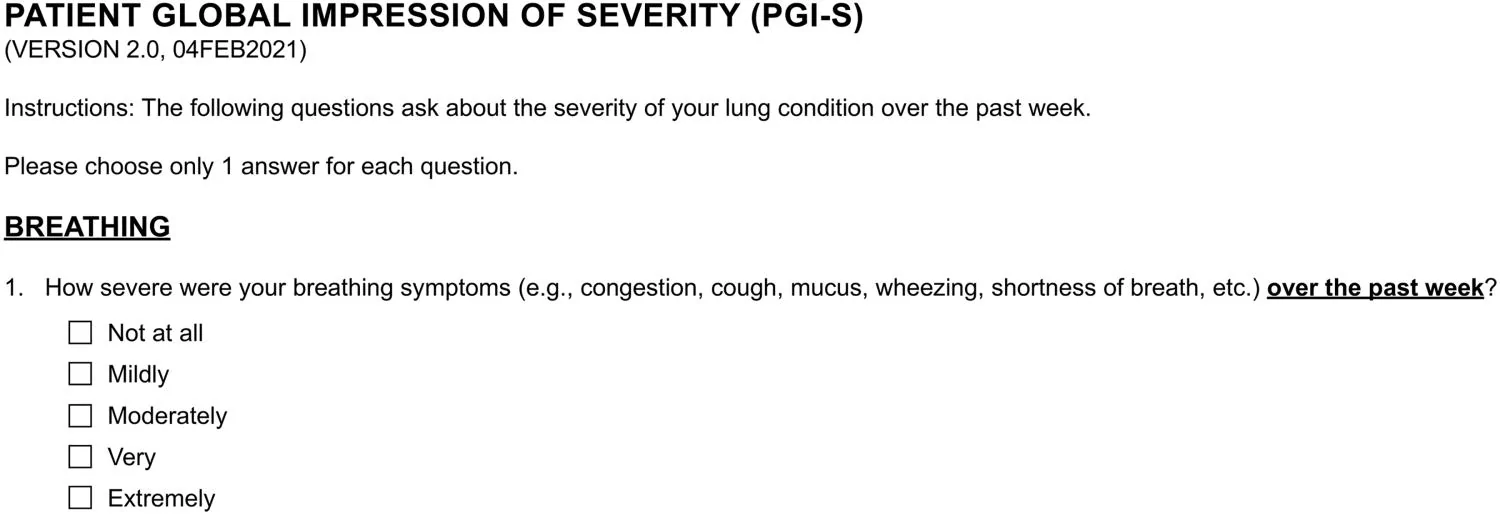
PGI-S response options. Abbreviation: PGI-S, Patient Global Impression of Severity.

To assess thresholds of clinically meaningful change within patients over time, anchor-based MWPC thresholds were estimated using improvement of 1 category on the PGI-S Respiratory (Figure 2) as the meaningful improvement anchor group. Mean and median change scores for the respiratory symptom score were computed between baseline and end of study (1-month off treatment) and supplemented with empirical cumulative distribution function (eCDF) curves to visualize the degree of separation between the anchor change groups at the location of the point estimate (i.e. median change score).

## Results

### Study population

The cross-sectional validation sample comprised 97 patients in ARISE and 265 patients in ENCORE; it excluded 2 patients in ARISE who were missing item-level QOL-B RD data. The longitudinal validation sample included all 99 patients enrolled in ARISE. Overall, 362 patients were included in the psychometric analysis; 70% were aged 65 years or older, 81% were female, and 69% were White (Table 1). Patients were enrolled between November 12, 2020, and January 25, 2024.

**Table 1.** Sample characteristics.

	Baseline sample (N = 364)^a^
Age group, n (%)	
< 65 years	111 (30.5)
≥ 65 years	253 (69.5)
Sex, n (%)	
Female	293 (80.5)
Male	71 (19.5)
Race, n (%)	
American Indian or Alaska Native	0 (0.0)
Asian	98 (26.9)
Black or African American	2 (0.6)
Native Hawaiian or Other Pacific Islander	0 (0.0)
White	250 (68.7)
Other	2 (0.6)
Multiple	0 (0.0)
Not reported	12 (3.3)
Unknown	0 (0.0)
Ethnicity, n (%)	
Hispanic or Latino	16 (4.4)
Not Hispanic or Latino	328 (90.1)
Not reported	16 (4.4)
Unknown	4 (1.1)
Weight at baseline, mean (min-max), kg	60 (34–139)
Height at baseline, mean (min-max), cm	164 (132–196)
BMI at baseline, mean (min-max), kg/m^2^	22.0 (13.6–42.9)
FEV_1_, mean (min-max), L	1.93 (0.55–4.22)

^a^ The baseline sample description is based on 364 patients, whereas the cross-sectional validation analysis sample at baseline comprised 362 patients due to 2 patients not providing any item-level data. Due to rounding, percentages may total more than 100%.

Abbreviations: BMI, body mass index; FEV_1_, forced expiratory volume in 1 second.

### Item-level descriptive assessments

Floor effects were present for 4 out of the 8 respiratory symptom score items (50%, Table S2). However, the items’ other response categories were reasonably endorsed by patients, supporting the items’ utility and relevance. Overall, 16 of the 28 unique inter-item correlations (57%) for the respiratory symptom score achieved |r| ≥ 0.4 (Table S3). One correlation (4%) exhibited r ≤ 0.2 and no inter-item correlations indicated item redundancy (i.e. r ≥ 0.8). Consequently, results indicate that the respiratory symptom score is largely composed of items expected to define a monolithic score, with varying strengths of association and no preliminary evidence of redundant items.

### Modern psychometric methods

An essentially unidimensional unit-weighted score appears empirically justified for the respiratory symptom score within this context of use. The 2-factor bifactor model satisfied the test of exact fit (RMSEA LCL = 0.000); the ratio between omega and hierarchical omega (0.773) was within rounding error of the threshold of 0.80, and H (0.847) met the criterion to support the total mean score (Table 2). The key assumptions of conditional independence and measurement invariance were tested and the total domain was found to be free from both local dependence and DIF/DTF. In the first stage of the employed DIF-Sweep procedure, no candidate DIF items were detected and no DIF severity assessment was required. The absence of DIF was corroborated with non–inference-based DTF evidence. For example, when examining DTF across age reference and focal groups, the Test D-Max indicated that the maximum observed score difference between older and younger populations was only 0.404 points (meaning that all other differences were numerically smaller across age groups in this sample), and the expected test score standardized difference of −0.05, interpreted like Cohen’s D statistics, indicated an absence of bias on the respiratory symptom score. Taken together, DIF and DTF evidence support the conclusion that the respiratory symptom score items and score are unbiased measures of respiratory symptoms.

**Table 2.** Modern psychometric methods: Model fit comparisons for 8-item respiratory symptom score.

	RMSEA (90% CI)	TLI	CFI	Omega	Hierarchical omega	Omega ratio	H-statistic
2-factor MIRT	0.090 (0.069, 0.111)	0.939	0.959	0.824	–	–	–
2-factor Bifactor	0.000 (0.000, 0.031)	1.008	1.000	0.905	0.699	0.773	0.847
3-factor MIRT	0.075 (0.052, 0.098)	0.958	0.974	0.807	–	–	–
3-factor Bifactor	0.000 (0.000, 0.021)	1.010	1.000	0.913	0.700	0.767	0.845

Abbreviations: CI, confidence interval; CFI, comparative fit index; MIRT, multidimensional item response theory; RMSEA, root mean squared error of approximation; TLI, Tucker-Lewis index.

### Reliability and validity

The respiratory symptom score demonstrated adequate internal consistency with a McDonald’s omega of 0.86 and a Cronbach’s alpha of 0.81 (Table 3). Corrected item-to-total correlations ranged between 0.66 to 0.84 and further supported a determination of acceptable internal consistency within this context of use, as no correlations fell below the prespecified success criterion of |r| ≥ 0.4.

**Table 3.** Reliability: Internal consistency estimates at baseline.

Item	McDonald’s omega (95% CI)	Cronbach’s alpha (95% CI)	Item-total polyserial correlation	Cronbach’s alpha if item is dropped
Domain score	0.86 (0.83, 0.88)	0.81 (0.78, 0.84)		
Chest congestion			0.84	0.77
Cough day			0.66	0.80
Cough mucus			0.68	0.79
SB greater activity			0.72	0.79
Wheeze			0.71	0.79
Chest pain			0.67	0.80
SB talking			0.75	0.78
Cough woken			0.74	0.79

Abbreviations: CI, confidence interval; SB, shortness of breath.

Test-retest reliability was estimated in a stable subset of 50 patients reporting no change in PGI-S Respiratory between screening and baseline. The ICC[2,1] estimate for the respiratory symptom score was 0.73. This exceeds the prespecified success criterion of ≥ 0.7, demonstrating the respiratory symptom score has acceptable test-retest reliability within this context of use.

The respiratory symptom score also demonstrated strong convergent validity (Table 4). As hypothesized, the respiratory symptom score had a moderate-to-strong association with each validator, whereby absolute correlations ranged between 0.51 and 0.77. The strongest association was observed between the respiratory symptom score and validators that measure respiratory symptoms: EXACT (r = −0.72), E-RS (r = −0.72), and SGRQ (r = −0.77). The direction of the correlation coefficient (negative or positive) is explained by scoring algorithms across measures.

**Table 4.** Concurrent validity at baseline.

	EXACT^a^	E-RS^a^	FACIT-Fatigue^b^	SGRQ^a^
Respiratory symptom score^b^	−0.72	−0.72	0.51	−0.77

^a^ EXACT, E-RS, and SGRQ: Higher score = higher severity, lower score = lower severity.

^b^ FACIT-Fatigue and respiratory symptom score: Higher score = lower severity, lower score = higher severity.

Abbreviations: E-RS, EXACT Respiratory Symptoms; EXACT, Exacerbations of Chronic Pulmonary Disease Tool; FACIT-Fatigue, Functional Assessment of Chronic Illness Therapy Fatigue Scale; SGRQ, St. George Respiratory Questionnaire.

Known-groups validity was demonstrated across PGI-S Respiratory severity groups (Table 5). Patients who responded “not at all” on the PGI-S Respiratory had a significantly higher respiratory symptom score (i.e. fewer respiratory symptoms) at baseline compared with those who selected “mildly,” “moderately,” or “very” (*P* < 0.001). Specifically, a sequentially ordered lower mean respiratory symptom score (i.e. more respiratory symptoms) was observed for the “mildly,” “moderately,” and “very” PGI-S Respiratory severity groups. Patients who responded “extremely” on the PGI-S Respiratory had a lower mean respiratory symptom score (indicating more respiratory symptoms) than those who selected “not at all,” “mildly,” or “moderately,” but had a higher score than those responding “very.” However, only 6 subjects responded “extremely,” so these results should be interpreted with caution.

**Table 5.** Known-groups validity at baseline.

PGI-S Respiratory reference category	PGI-S Respiratory effect category	N	LSM estimate (95% CI)	LSM contrast	*P*-value	Semi-partial omega squared (95% CI)
Not at all		35	83.10 (78.83, 87.36)			
	Mildly	142	70.89 (68.78, 73.01)	12.20	< 0.001	0.06 (0.02, 0.12)
	Moderately	128	58.85 (56.62, 61.08)	24.24	< 0.001	0.21 (0.14, 0.28)
	Very	51	40.69 (37.15, 44.22)	42.41	< 0.001	0.39 (0.31, 0.45)
	Extremely	6	47.22 (36.93, 57.52)	35.87	< 0.001	0.10 (0.05, 0.16)

Abbreviations: CI, confidence interval; LSM, least squares means; PGI-S, Patient Global Impression of Severity.

### Meaningful change

Prior to the MWPC analysis, Pearson correlations between respiratory symptom score and PGI-S Respiratory change scores at end of study (1-month off treatment) were calculated. A correlation of −0.52 indicated that the PGI-S Respiratory was a suitable anchor for use in these analyses.^35^ Note that the correlation was negative because for the QOL-B RD, higher scores indicate better symptoms and for the PGI-S Respiratory, higher responses indicate worse symptom severity.

**Figure 3.**
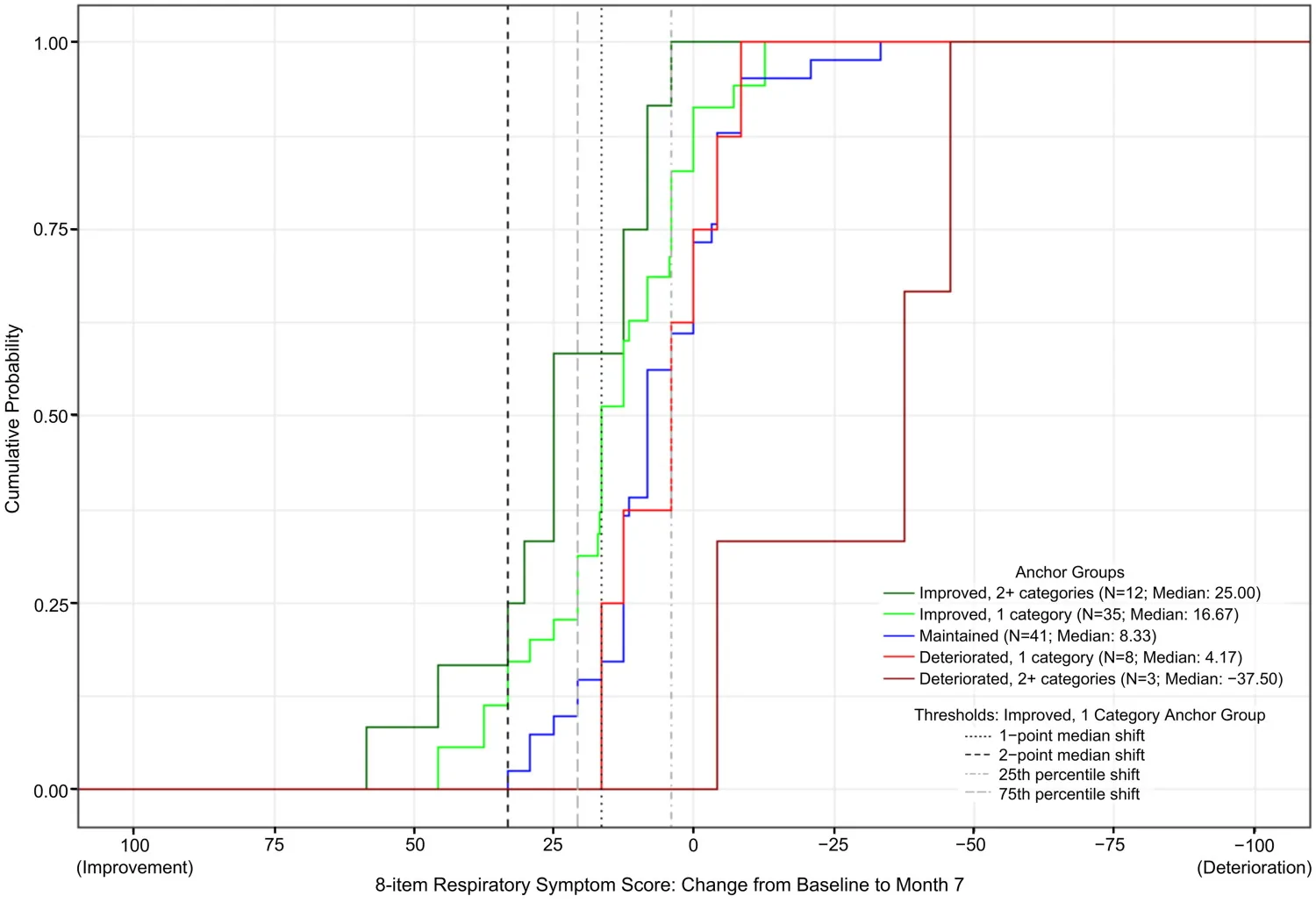
eCDF of change in respiratory symptom score from baseline to end of study by PGI-S Respiratory anchor group (N = 99 ARISE patients)^a^. ^a^End of study: 1 month off treatment (month 7). Abbreviations: eCDF, empirical cumulative distribution function; PGI-S, Patient Global Impression of Severity.

The anchor-based MWPC analyses supported a 16.67-point median increase from baseline (95% CI: 8.33–16.83 points) as the estimated threshold of clinically meaningful within-patient improvement for the respiratory symptom score (Table 6). The eCDF curves showed clear and consistent separation between the improved and maintained anchor groups across all changes in the respiratory symptom score, including the estimated 16.67 MWPC threshold (Figure 3).

**Table 6.** Anchor-based mean and median change from baseline in respiratory symptom score at end of study (N = 99 ARISE patients)^a^.

Thresholds by anchor group			
Anchor group	Mean (95% CI)	Median (95% CI)	N (%)
Improved, 2+ categories	24.04 (13.67, 34.42)	25.00 (8.33, 30.17)	12 (12.1)
Improved, 1 category	14.92 (9.86, 19.98)	16.67 (8.33, 16.83)	35 (35.4)
Maintained	6.11 (1.99, 10.23)	8.33 (0.00, 12.50)	41 (41.4)
Deteriorated, 1 category	5.21 (−2.64, 13.05)	4.17 (−8.33, 16.67)	8 (8.1)
Deteriorated, 2+ categories	−29.17 (NA)^b^	−37.50 (NA)^b^	3 (3.0)

^a^ End of study: 1 month off treatment (month 7).

^b^ Sample size did not permit calculation of confidence intervals.

Abbreviations: CI, confidence interval; NA, not applicable.

## Discussion

Evidence presented in this manuscript builds upon previous qualitative research on the content validity of the QOL-B RD in patients with MAC lung disease.^14^ The psychometric validation results show that the 8-item respiratory symptom score derived from the QOL-B RD has robust structural validity and is an unbiased, reliable, and valid PRO tool able to measure respiratory symptoms and detect meaningful within-patient change in respiratory symptoms in adult patients with a new or recurrent diagnosis of MAC lung disease.^i^ The modern psychometric scoring statistics supported an essentially unidimensional unit-weighted mean respiratory symptom score in MAC lung disease.

Readers primarily familiar with classical test theory typically seek clarity on the concept of essential versus absolute unidimensionality. For an explanation of the statistical methods underpinning essential dimensionality, a detailed review of the literature and explanation of how these methods are applied in modern psychometric methods is given in Supplementary Appendix A. Briefly, achieving criteria satisfying essential unidimensionality supports the determination, according to Stout (1990), that “minor or idiosyncratic dimensions should be ignored in assessing test dimensionality from the applications viewpoint”.^27^ Following Rodriguez et al.,^29^ when the ratio of the internal consistency based only on the general factor in a bifactor to internal consistency based on all factors in a bifactor exceeds 0.8 then out of all sources of variance in the unit-weighted total score the general factor is dominant, accounting for 80% of the explainable internal consistency. In such a circumstance, the unit-weighted total score is essentially unidimensional. Thus, essential unidimensionality does not require an omega ratio of 1, which corresponds to absolute unidimensionality.^27^ Because our estimated omega ratio (0.773) is within rounding error of 0.8, treating the 8-item respiratory symptom score as essentially unidimensional is empirically justified and similar to applications that have treated the QOL-B RD as a unidimensional score per the developer’s instructions.

In our analysis of the 8-item respiratory symptom score, 4 items demonstrated baseline floor effects. For the wheezing, chest pain, dyspnea while talking, and awakened due to cough items, 47.38%, 48.21%, 35.26%, and 36.36% of respondents endorsed the “never” category, respectively (Table S2). While a previous validation study by Henkle et al. in MAC lung disease identified no floor or ceiling effects in the QOL-B RD items,^36^ we hypothesize both “wheezing” and “chest pain” would be expected to be preponderated with “never” responses given their intermittent or heterogeneous presentation within a MAC lung disease population. Regardless, such floor effects are routinely cited as a risk to improvement detection. While this can be true, the risk depends on factors like the number of items with such floor effects and the nature of the floor effect. Moreover, there is a need to consider maintaining content validity of the instrument along with the strong countervailing effect of the other items not exhibiting this pattern and how these items in concert combine to define a score that may still be sensitive. In fact, what we see in this data is not an attenuation in the meaningful improvement thresholds. Rather, our estimated anchor-based meaningful change threshold associated with minimal improvement is greater than the degree of change historically observed and reported, as described below. We therefore believe that the superficial appearance of floor effects in 4 items at baseline had no meaningful effect and is not a cause for concern.

Our estimated anchor-based meaningful change threshold would require a 16.67-point improvement to achieve clinically meaningful within-patient improvement for the 8-item respiratory symptom score. This estimate is aligned with our findings for the 9-item QOL-B RD score (median: 14.81-point increase from baseline; 95% CI: 8.82–18.52 points; Table S11) and could be used in MAC lung disease clinical trials to evaluate changes in symptomology with treatment. Of note, this anchor-based estimate is greater than previously reported minimal important differences for the 9-item QOL-B RD, including the distribution-based estimate of 6.4 to 6.9 points in patients with MAC lung disease reported by Henkle et al.,^36^ and the 8.0-point estimate in patients with bronchiectasis and gram-negative infection reported by Quittner et al.,^13^ which was the average of two distribution-based methods and one anchor-based method.

A variety of factors may explain why our meaningful change threshold estimate (+16.67) is larger than those previously reported. First, the extant threshold estimates cited above were either based entirely (Henkle et al.) or largely (Quittner et al.) on distribution-based methods. The FDA no longer considers distribution-based estimates interpretable or patient-centric.^37^ Broadly, distribution-based estimates tend to be smaller than anchor-based estimates. Second, our symptom-specific anchor, recommended by the FDA’s guidance for interpretable and patient-centric estimates,^37^ has never been used in any research other than this research and therefore definitionally would be expected to yield estimates distinct from those previously estimated via either distribution-based methods or less-specific or different anchors. Finally, our study sample was patients with incident MAC lung infection treated with active antibiotic therapy, including 50% of the sample receiving amikacin liposome inhalation suspension (ALIS). Consequently, our estimated threshold should not and cannot be expected to be consistent with prior estimates derived via different methods in different contexts of use, and under different treatment regimens.

The present psychometric validation analysis had several strengths and limitations. It included a large study sample from geographically diverse multi-center clinical trials that was majority White, female, and aged ≥ 65 years, which is representative of adults with MAC lung disease.^38^ There was no evidence of systematic bias across age, sex, or MAC history groups in the DIF or DTF results, although sample sizes less than 200 per group can pose challenges to DIF,^39–41^ and our total analysis sample of 362 patients was below this boundary. In addition, test-retest reliability was satisfactory in this analysis, although the test-retest interval was up to 70 days (ideal range: 2–3 weeks). However, MAC are very slow-growing organisms, and thus MAC lung disease tends to be a slowly progressing condition over a period of months or years rather than days, particularly among individuals with noncavitary lung disease.^42^ Thus, based on the natural history of MAC lung disease, a test-retest reliability interval of 1 to 2 months is not expected to result in significant changes in respiratory symptoms attributable to this disease. The validation analysis followed a prespecified analysis plan developed by the first author independent of the sponsor. The ARISE and ENCORE investigational site staff, patients, and sponsor representatives were all blinded to post-baseline sputum culture data as well as treatment assignment in order to minimize potential bias. All PROs are subjective, so in addition to the blinding described above, patients did not have access to their previously completed PRO responses in order to minimize potential PRO response bias. Finally, the patients in ARISE and ENCORE had not started treatment for their current MAC lung infection at enrollment, so future work should examine the respiratory symptom scale score among treatment-experienced patients.

The psychometric evidence provides preliminary support for the use of the respiratory symptom score as an endpoint to evaluate subjective respiratory symptoms in patients with MAC lung disease. Development of this fit-for-purpose PRO endpoint for the MAC lung infection context of use was based on current best practice and regulatory guidance. Our novel analysis of the 8-item respiratory symptom scale is the first to use anchor-based estimation of meaningful change thresholds with a symptom-specific anchor, as recommended in the FDA’s guidance for interpretable and patient-centric estimates. This PRO tool could be used in clinical trials to complement endpoints such as sputum cultures or imaging to help establish an evidence-based therapeutic plan to support adequate treatment periods. Additional research is needed to evaluate whether treatment of MAC lung disease results in a meaningful improvement in respiratory symptoms as measured by the respiratory symptom score.

## Conclusions

The results of this psychometric validation analysis demonstrate the 8-item respiratory symptom score derived from the QOL-B RD has adequate reliability, validity, and responsiveness for assessing respiratory symptoms in adults with a new or recurrent diagnosis of MAC lung disease. The respiratory symptom score is suitable for use in clinical trials for evaluating the impact of treatment on respiratory symptoms of MAC lung disease.

## Supplementary Material

Supplemental Material

## Note

i Psychometric evaluation of the 9-item QOL-B RD, as presented in the online Supplemental Material, yielded similar results to that of the 8-item respiratory symptom score.

## Transparency

### Declaration of financial/other relationships

Kevin C. Mange, Mariam Hassan, Marie-Laure Nevoret, and Dayton W. Yuen are employees and shareholders of Insmed Incorporated. Daniel Serrano, Shauna McManus, Lauren Podger, Sam Parsons, and Bryant Barnes were employees of OPEN Health Group at the time of study, which was funded by Insmed Incorporated. Charles L. Daley reports grant support, advisory board fees, and consulting fees from Insmed Incorporated. Dr. Daley also reports grant support from AN2 Therapeutics, Bugworks, Paratek Pharmaceuticals, Juvabis; advisory board work with AN2 Therapeutics, AstraZeneca, Cepheid, Galapagos, Hyfe, MannKind, Matinas Biopharma, MicuRx, NobHill, Spero Therapeutics, Zambon; consulting with Genentech, Pfizer; data monitoring committee work with Otsuka Pharmaceutical, and Bill and Melinda Gates Foundation.
