# Supplementary material for: Validation of a respiratory symptom score from the Quality of Life-Bronchiectasis Questionnaire in adults with Mycobacterium avium complex lung disease
Source: Patient Relat Outcome Meas. 2026 Apr 28;17(1):4–15. doi: 10.1080/1179271X.2026.2655469 (PMC13536775; doi:10.1080/1179271X.2026.2655469)
Supplement: Supplementary file 1 [file DPRO_A_2655469_SM9973.docx]

Validation of a Respiratory Symptom Score from the Quality of Life-Bronchiectasis Questionnaire in Adults with *Mycobacterium avium* Complex Lung Disease

Daniel Serrano, PhD, Mariam Hassan, PhD, B.Pharm, Marie-Laure Nevoret, MD, Dayton W. Yuen, PharmD, Shauna McManus, MPH, Lauren Podger, MSc, Sam Parsons, DPhil, Bryant Barnes, BS, Charles L. Daley, MD, Kevin C. Mange, MD, MSCE

Supplemental Material

Section 1. Supplemental Methods

Section 2. Supplemental Results: 8-item Respiratory Symptom Score

Section 3. Psychometric Validation of the 9-item QOL-B RD

Appendix A. Essential Dimensionality Review

Section 1. Supplemental Methods

Study Design

Data were analyzed from the ARISE (NCT04677543) and ENCORE (NCT04677569) clinical trials (see **Figure 1** in main text), which were conducted concurrently and had identical eligibility criteria (**Table S1**) [1,2]. Briefly, eligible patients were adults (≥ 18 years) with non-cavitary lung disease who had a current diagnosis (initial or subsequent [i.e., second or third]) of MAC lung infection and had not started treatment for their current MAC lung infection. Patients who were candidates for the studies were randomly assigned to either trial at the time of informed consent until ARISE was fully enrolled.

In both trials, study participants were randomized to amikacin liposome inhalation suspension (ALIS) 590 mg or empty liposome control once daily in combination with a background regimen of azithromycin 250 mg and ethambutol 15 mg/kg tablets once daily. In ARISE, treatment was administered continuously for 6 months, followed by PRO evaluations and end of study evaluation at month 7 (1 month off treatment) (**Figure 1**). In ENCORE, treatment was administered continuously for 12 months, followed by PRO evaluations at month 13 (1 month off treatment) and month 15 (end of study; **Figure 1**).

The primary objective of the ARISE study was to generate evidence to support the validation of the Quality of Life-Bronchiectasis respiratory domain (QOL-B RD) and the Patient-Reported Outcomes Measurement Information System Short Form v1.0 – Fatigue 7a (PROMIS-F SF-7a) for use in adult patients with MAC lung disease [1]. Psychometric validation of the PROMIS-F SF-7a has been published separately [3]. The primary objective of ENCORE was to evaluate the efficacy of ALIS plus background regimen versus empty liposome control plus background regimen on patient-reported respiratory symptoms at month 13 [2]. Both studies were conducted in accordance with the Declaration of Helsinki. Local institutional review boards or independent ethics committees approved the study protocols. All patients provided written or electronic informed consent.

Table S1. Principal ARISE and ENCORE Inclusion and Exclusion Criteria

| Inclusion criteria | Male or female, ≥ 18 years of age (19 years or older in South Korea, 20 years or older in Japan)  Current diagnosis of MAC lung infection. MAC or mixed infection with MAC as the dominant species is allowed, with MAC as the intended organism for treatment  Positive sputum culture for MAC within 6 months prior to Screening  Positive sputum culture for MAC at Screening  A chest CT scan, read locally, within 6 months prior to Screening to determine presence and size of pulmonary cavities. Participants who do not have a chest CT scan within 6 months prior to Screening will be required to obtain a chest CT scan, read locally, during Screening  In the Investigator’s opinion, documented respiratory signs/symptoms at Screening that are attributable to the current MAC lung infection  An average QOL-B respiratory domain score of ≤ 85 based on scores at Screening and on the day of enrollment prior to randomization  In the Investigator’s opinion, underlying lung disease (e.g., COPD, bronchiectasis) has been managed according to best local standard of care, and on stable maintenance therapy for a minimum of 4 weeks prior to randomization  Willingness and ability to adhere to prescribed study treatment during the study  Ability to produce (spontaneously or with induction) approximately 2 mL of sputum for mycobacteriology at Screening  WOCBP (i.e., fertile following menarche and until becoming post-menopausal unless permanently sterile) and fertile men (i.e., all men after puberty unless permanently sterile by bilateral orchidectomy) agree to practice a highly effective method of birth control from Day 1 to at least 90 days after the last dose  Provide signed informed consent prior to administration of study drugs or performing any study related procedure  Be able to comply with study drugs use, study visits, and study procedures as defined by the protocol  Men with partners who are WOCBP (pregnant or non-pregnant) agree to use condoms and non-pregnant partners should practice a highly effective method of birth control |
| --- | --- |
| Exclusion criteria | Diagnosis of cystic fibrosis  History of more than 3 MAC lung infections  Received any mycobacterial antibiotic treatment for current MAC lung infection  Refractory MAC lung infection, defined as having positive MAC cultures while being treated with a multidrug mycobacterial antibiotic treatment regimen for a minimum of 6 consecutive months and no documented successful treatment, defined as negative sputum culture for MAC and cessation of treatment  Relapse of prior MAC lung infection, defined as positive sputum culture for MAC ≤ 6 months of cessation of prior successful treatment  Evidence of any pulmonary cavity ≥ 2 cm in diameter, as determined by chest CT scan, read locally, within 6 months prior to Screening  Radiographic finding of new lobar consolidation, atelectasis, significant pleural effusion, or pneumothorax during routine clinical care within 2 months prior to Screening  Active pulmonary malignancy (primary or metastatic) or any malignancy requiring chemotherapy or radiation therapy within 1 year prior to Screening or anticipated during the study  Acute pulmonary exacerbation (e.g., COPD or bronchiectasis) requiring treatment with antibiotics, or corticosteroids (IV or oral), within 4 weeks prior to and during Screening  Current smoker  History of lung transplantation  Prior exposure to ALIS (including clinical study)  Known hypersensitivity or contraindications to use to ALIS, aminoglycosides, or any of their excipients  Disseminated MAC infection  Administration of any investigational drug within 8 weeks prior to Screening  Known or suspected acquired immunodeficiency syndromes (HIV-positive, regardless of CD4 counts). Other immunodeficiency syndromes that may interfere with study participation in the opinion of the Investigator  Current alcohol, medication, or illicit drug abuse  Known and active COVID-19 infection  Known hypersensitivity or contraindications to use to ethambutol, azithromycin (including other macrolides or ketolides), or any of their excipients per local labeling guidance |

Abbreviations: ALIS, amikacin liposome inhalation suspension; COPD, chronic obstructive pulmonary disease; CT, computed tomography; IV, intravenous; MAC, *Mycobacterium avium* complex; WOCBP, women of child-bearing potential

PRO Assessments

The QOL-B is a self-administered instrument that assesses symptoms, functioning, and health-related quality of life for persons living with non-cystic fibrosis bronchiectasis [4]. It contains 37 items divided into 8 domains. Each item is scored from 1 to 4, with the domain score standardized on a 0- to-100-point scale, with higher scores representing fewer symptoms. The entire QOL-B questionnaire was administered to all study participants, and the individual domains were scored according to the rights granted by the copyright holder. In addition to the 9-item QOL-B respiratory domain (QOL-B RD) score, an 8-item respiratory symptom score removing the QOL-B RD sputum color item (item 32) was explored because sputum color is a clinical sign rather than a symptom exemplifying how a patient may feel or function, and item 32 demonstrated weaker endorsement, relevance, and meaningfulness compared to other QOL-B RD items in previous qualitative research in patients with MAC lung disease [5]. The 8 items included in the respiratory symptom score were identified as most appropriate to measure clinical outcomes in the target population of MAC lung disease. The Patient Global Impression of Severity (PGI-S) Respiratory served as the primary anchor for the analysis of meaningful within-patient change (MWPC) in the respiratory symptom score because it assesses the same concept of interest (respiratory symptoms), is easily interpretable in patients with MAC lung disease, and has the same recall period (past week) as the QOL-B [6].

ARISE participants completed the PRO assessments at pre-specified in-clinic visits (**Figure 1**). For both studies, the PRO instruments were provided in electronic format on a computer tablet and conducted at approximately the same time of day at the specified timepoints. All PRO assessments were completed prior to other study assessments (e.g., physical examination, vital signs, laboratory assessments, audiology) and administration of study drugs.

Statistical Software

Response patterns for the respiratory symptom score items were generated using the xtabs procedure in R [7]. Inter-item correlations were estimated using polychoric correlations obtained from version 2.3.3 of the psych package in R [8].

Item exploratory factor analyses, item response theory (IRT) models, local dependence, and differential item functioning (DIF) were evaluated using the mirt R package, version 1.39 [9].

Internal consistency was estimated using McDonald's omega and Cronbach’s alpha, which were generated using the psych package in R, version 2.3.3 [8]. These estimates were augmented with item-to-total correlations estimated via polyserial correlations using the polycor package, version 0.8.1 [10].

Estimates of test-retest reliability were based on the two-way mixed effects intraclass correlation coefficient (ICC[2,1]) [11], which was estimated using the irr package, version 0.84.1 [12].

The lm procedure embedded within base R [7] as well as the emmeans package, version 1.8.7 [13], and the effectsize package, version 0.8.3 [14], were used in the known-groups validity evaluation.

Modern Psychometric Methods

Item exploratory factor analysis (iEFA) was conducted to determine the number and item composition of domains measured by the respiratory symptom score items. Samejima’s graded response model (GRM) was used for all items [15]. The GRM is the latent-variable analog of a multivariate proportional odds model. The optimal number of factors for the respiratory symptom score items was determined based on model fit indices. Model fit was evaluated using the C2-based $\chi^{2}$ and root mean squared error of approximation (RMSEA). RMSEA model fit criteria were based on the 90% lower confidence limits (LCL) corresponding to the classical tests of exact fit (LCL = 0) or close fit (LCL < 0.05) [16]. The test of close fit was more reasonable than the expectation of exact model fit and considered acceptable given exact fit failure. The Tucker-Lewis Index (TLI) and comparative fit index (CFI) were also employed in model fit evaluations; acceptable model fit for TLI and CFI corresponded to values ≥ 0.95 [17].

Item response theory (IRT) models were used to characterize the structural validity of the respiratory symptom score based on the iEFA-detected domains. IRT models tested the quality and precision of the respiratory symptom score items as well as item redundancy and bias. In addition, dimensionality and scoring statistics were employed to empirically determine the optimal scoring procedure for the respiratory symptom score (i.e., unit-weighted versus empirically-weighted and essentially unidimensional [18] versus multidimensional). The IRT models were estimated using heterogeneous likelihoods using the same models estimation detailed for iEFA. To assess essential dimensionality, multidimensional IRT (MIRT) and bifactor models were compared. The same model fit indices and success criteria used for iEFA were used to select the optimal IRT model. Dimensionality statistics used to evaluate essential dimensionality and scoring included McDonald's omega ratios and the H statistic [19]. Essential unidimensionality of the respiratory symptom score data was supported if the ratio of all modellable sources explaining variance in a unit-weighted total score ($\omega$) to that variance in a unit-weighted total score explained by a total domain ($\omega_{H}$), $\omega_{H}/\omega$, was equal to or greater than 0.80. Such an omega ratio for a total domain obtained from a bifactor model would indicate that 80% of the variance in a unit-weighted total score would be accounted for by the total domain, empirically justifying an essentially unidimensional unit-weighted total score [20]. Corroborating evidence supporting a unit-weighted essentially unidimensional respiratory symptom score would come from an H value ≥ 0.70 [20].

Item redundancy and the assumption of conditional independence were evaluated by identifying local dependence (LD) among items by computing the Chen and Thissen’s G^2^ statistic [21], with any item demonstrating a significant marginal χ^2^ or a G^2^ value higher than 3 indicating problematic LD. Inferential tests employed in LD analyses were multiplicity-adjusted using the Benjamini-Hochberg false discovery rate (FDR) adjustment [9].

DIF Assessment

Differential item functioning analysis was conducted using the Langer-improved Wald-2 DIF sweep procedure [22] to determine whether 3 populations of interest responded to the respiratory symptom score items differentially: younger versus older participants (above or below median age), male versus female participants, and participants with an initial versus subsequent MAC lung infection.

In stage 1 of the Wald-2 DIF Sweep, item parameter estimates were constrained to be the same across the groups and structural parameters (i.e., mean and variance of the latent distribution within the focal group) were estimated. A second IRT model was also estimated, in which the structural parameters for the focal group were held fixed at their previously estimated values and the item parameters of both groups were freely estimated. The Wald test with Benjamini-Hochberg FDR p-value adjustment was used to determine which items did not display DIF [23]. The non-DIF items constituted the set of “anchor” items used in stage 2, whereas the remaining items were targets for formal DIF assessment in stage 2.

In stage 2, item parameters corresponding to the anchor items were held fixed across focal and reference groups, the structural parameters of the focal group were freely estimated, and the item parameters for the suspected DIF items were freely estimated across the focal and reference groups. Using these estimation methods in stage 2 allowed us to test whether item parameters significantly differed between groups, adjusting for the structural parameter differences across groups. Therefore, stage 2 tests for DIF while matching on latent score values to determine if given the same score, whether the focal and reference groups systematically respond differently to a given item. In theory, they should not; if they do, then systematic item bias exists across groups. A second and final Wald test with a Benjamini-Hochberg FDR p-value adjustment was then used to determine which of the suspected DIF items identified in stage 1 presented with significant DIF after formal testing in stage 2. A DIF severity assessment was conducted in which any items that demonstrated significant DIF in the formal assessment of stage 2 were scrutinized further to determine whether significant DIF was severe or not.

In cases where no DIF exists, all items will present as “anchor” items in the first stage of the DIF-sweep, and the second stage is obviated because it is inestimable under this procedure. The conclusion in such an outcome is that no DIF exists and no DIF severity assessment is possible. However, sample sizes less than 200 per group can pose challenges to DIF that can be compounded by asymmetries in reference and focal group size [24-27].

DIF severity was evaluated based on the procedure employed in Hansen et al. [28]. Expected score curves were plotted for any candidate items identified in the final stage of the DIF sweep, stratified by reference and focal group. Expected score curves provide a visualization of how a given level of the underlying trait being measured (represented as θ) is related to average response, given the probability of responding in each possible response category. The probability of responding in each response category was dependent on the item parameters from the models generated during the final stage of the DIF sweep. If the expected score curves for each group was similar, that is, if there is no range of θ where one group would be expected to have a different response than the other group, DIF for the given item was considered ignorable. By contrast, if there was substantially distinct expected score curves for the reference and focal groups, then DIF was considered to be severe and non-ignorable. In this instance, the weighted area between curves (wABC) statistic, which measures the area between the two expected score curves of the focal and reference groups. would be computed for these items only. DIF severity was defined by wABC exceeding 0.30 [28]. If the DIF is sufficiently severe that scores were affected, then items would either be deleted (if homogeneity is prioritized) or differential scoring would be implemented across reference and focal groups (if inclusion of items was prioritized).

Provisional evidence of any candidate items with identified DIF associated with an wABC exceeding 0.30 was augmented with non-inferential tests of differential test functioning (DTF). Differential test functioning is particularly useful, as how an individual item behaves is often less important than how a score behaves across populations. For example, an individual item may display DIF, but not affect QOL-B RD scores. Specifically, the expected test score standardized difference (ETSSD), a Cohen’s D analog for test score differences, interpreted following Cohen’s rules and the maximum expected test score difference (Test D-Max) was computed [29-31]. A Test DMax less than one point demonstrated acceptable DTF [30,31]. Additionally, findings were corroborated by plotting the test scoring functions stratified by the same DIF reference and focal categories.

Test-Retest Reliability

Test-retest reliability was estimated in a stable subset of patients reporting no change (i.e., change score of zero) in PGI-S Respiratory between screening and baseline (up to 70-day retest interval). This interval was longer than typical of test-retest reliability testing because patients could not be randomized (Baseline visit) until final MAC culture results were available from sputum samples collected at Screening, which may take up to 8 weeks due to the slow-growing nature of NTM. A shorter interval subset analysis was not feasible due to the small sample size.

Imputation

The handling of missing data and appropriate imputations were performed after all efforts failed to obtain the data. If appropriate, retrieved data were used. A retrieved assessment was defined as an assessment collected after the early discontinuation of ALIS/ELC; a retriever was defined as a patient who stayed in the study and continued to provide data after discontinuation of the randomized treatment regimen.

Missing individual PRO items were not imputed. Missing PRO domain scores at Month 7 were imputed using the multiple imputation method, which is appropriate to appropriately estimate the mean and variance of the treatment effect.

The influence of intercurrent events (ICEs) and the presence of the missing data on the estimation of change from baseline to Month 7 PRO means were taken into consideration. The ICEs were defined as post-randomization events leading to early ALIS/ELC therapy termination due to death, ALIS/ELC-related TEAE, use of rescue medication, or lack of efficacy.

The following considerations were included:

1. A patient who died after randomization would be assigned the worst change score in the entire sample as the change from baseline to Month 7
2. Data for patients who completed 6 months of the randomized treatment but their Month 7 PRO was not available were imputed based on the observed status distribution from the patients who completed 6 months of randomized treatment, had Month 7 data available, and were randomized to the same treatment group. This approach is consistent with a missing at random (MAR) approach.
3. When retrieved assessments were unavailable, the missing data were imputed as follows:

- For patients experiencing ICEs other than death, their missing Month 7 PRO data were imputed based on the retrieved assessment distribution (flagged as Ref_MNAR) from those retrievers who experienced ICE and are exposed to an altered treatment regimen after their ICEs. MNAR imputation of the data for those subjects experiencing ICE was applied.
- For patients not experiencing ICE, their missing Month 7 PRO data were imputed based on the observed data distribution (flagged as Ref_MNAR) of patients who completed 6 months of randomized therapy, had Month 7 PRO data assessment available, and were randomized to the same treatment group. This approach is consistent with a MAR approach.

The missing data were multiply imputed using PROC MI in SAS. Fifty replications were sufficient to reflect variability of the imputations. The variables used in the MI model (predictors) were randomization stratum (history of MAC lung disease, initial or subsequent infection), PRO score at baseline, and treatment group. The FCS option was used, allowing a joint distribution of continuous and categorical variables. To avoid imputed values being outside of range, the REGPREDMEANMATCH option was used. This method specifies the predictive mean matching method for continuous variables. It imputes a value randomly from a set of observed values whose predicted values are closest to the predicted value for the missing value from the specified model. The fixed seed, 68763497 (generated randomly), was used to enable reproducible results.

Following each round of imputation, a full set of PRO domain scores at Month 7 was created by combining the observed values and sets of imputed values under the MNAR and MAR assumptions. This process resulted in a total of 50 full sets, which were averaged together for each subject to generate their total PRO domain score at Month 7.

Section 2. Supplemental Results: 8-item Respiratory Symptom Score

Table S2. Item Response Pattern: 8-item Respiratory Symptom Score

| **Item** | **N (%) of Participants** | | | | |
| --- | --- | --- | --- | --- | --- |
|  | **A Lot** | **A Moderate Amount** | **A Little** | **Not at All** | **Missing** |
| Have you felt congestion in your chest? | 43 (11.85) | 103 (28.37) | 154 (42.42) | 62 (17.08) | 1 (0.28) |
| Have you been coughing during the day? | 53 (14.6) | 117 (32.23) | 172 (47.38) | 20 (5.51)^a^ | 1 (0.28) |
| Have you had to cough up mucus? | 51 (14.05) | 128 (35.26) | 154 (42.42) | 29 (7.99)^a^ | 1 (0.28) |
|  | **Always** | **Often** | **Sometimes** | **Never** | **Missing** |
| Have you had shortness of breath with greater activity, such as housework or yardwork? | 59 (16.25) | 92 (25.34) | 160 (44.08) | 51 (14.05) | 1 (0.28) |
| Have you had wheezing? | 3 (0.83)^a^ | 30 (8.26)^a^ | 157 (43.25) | 172 (47.38)^b^ | 1 (0.28) |
| Have you had chest pain? | 7 (1.93)^a^ | 26 (7.16)^a^ | 154 (42.42) | 175 (48.21)^b^ | 1 (0.28) |
| Have you had shortness of breath when talking? | 9 (2.48)^a^ | 60 (16.53) | 165 (45.45) | 128 (35.26)^b^ | 1 (0.28) |
| Have you woken up during the night because you were coughing? | 12 (3.31)^a^ | 43 (11.85) | 175 (48.21) | 132 (36.36)^b^ | 1 (0.28) |

^a^ Sparseness. ^b^ Floor effect.

Table S3. Inter-Item Polychoric Correlations at Baseline: 8-item Respiratory Symptom Score Analysis

| **Item** | **Chest Congestion** | **Cough Day** | **Cough Mucus** | **SB Greater Activity** | **Wheeze** | **Chest Pain** | **SB Talking** | **Cough Woken** |
| --- | --- | --- | --- | --- | --- | --- | --- | --- |
| Chest Congestion | 1.00 |  |  |  |  |  |  |  |
| Cough Day | 0.53 | 1.00 |  |  |  |  |  |  |
| Cough Mucus | 0.52 | 0.57 | 1.00 |  |  |  |  |  |
| SB Greater Activity | 0.46 | 0.19^a^ | 0.24 | 1.00 |  |  |  |  |
| Wheeze | 0.54 | 0.21 | 0.32 | 0.45 | 1.00 |  |  |  |
| Chest Pain | 0.48 | 0.26 | 0.21 | 0.43 | 0.48 | 1.00 |  |  |
| SB Talking | 0.49 | 0.24 | 0.26 | 0.73 | 0.51 | 0.46 | 1.00 |  |
| Cough Woken | 0.57 | 0.49 | 0.51 | 0.32 | 0.39 | 0.37 | 0.36 | 1.00 |

^a^ Correlation ≤ 0.2.

Abbreviation: SB, shortness of breath.

Section 3. Psychometric Validation of the 9-item QOL-B RD

The results of the psychometric evaluation for the 9-item QOL-B RD are presented herein. Generally, the same methods were used for the 9-item analysis as for the 8-item respiratory symptom score analysis; exceptions are described below. Blinded data were analyzed using R statistical software [7].

Analysis Samples

For the 9-item QOL-B RD analyses, the cross-sectional validation sample comprised data from 229 patients. This included screening and baseline data from 97 patients in ARISE who provided item-level QOL-B RD data at baseline, as well as baseline data from the first 132 patients randomized into ENCORE. Item-level QOL-B RD data were missing for 2 patients in the ARISE study. For the cross-sectional validation analysis sample, most patients were aged 65 years or older (67.8%), White (73.0%), and female (80.9%; **Table S4**).

The longitudinal validation analysis sample comprised all 99 patients enrolled in ARISE; QOL-B RD raw scores were imputed for those missing item-level data (see **Supplementary Materials,** **Section 1**).

Table S4. Sample Characteristics: 9-item QOL-B RD Analysis

|  | **Baseline Sample (N=231)^a^** |
| --- | --- |
| Age group, n (%) |  |
| < 65 years | 75 (32.6) |
| ≥ 65 years | 156 (67.8) |
| Sex, n (%) |  |
| Female | 186 (80.9) |
| Male | 45 (19.6) |
| Race, n (%) |  |
| American Indian or Alaska Native | 0 (0.0) |
| Asian | 55 (23.9) |
| Black or African American | 1 (0.4) |
| Native Hawaiian or Other Pacific Islander | 0 (0.0) |
| White | 168 (73.0) |
| Other | 0 (0.0) |
| Multiple | 0 (0.0) |
| Not reported | 6 (2.6) |
| Unknown | 1 (0.4) |
| Ethnicity, n (%) |  |
| Hispanic or Latino | 11 (4.8) |
| Not Hispanic or Latino | 214 (93.0) |
| Not reported | 6 (2.6) |
| Weight at baseline, mean (min-max), kg | 60.16 (36.0-139.0) |
| Height at baseline, mean (min-max), cm | 164.79 (132.1-195.6) |
| BMI at baseline, mean (min-max), kg/m^2^ | 22.02 (13.6-42.0) |
| FEV_1_, mean (min-max), L | 1.97 (0.55-4.22) |

^a^ The baseline sample description is based on 231 patients, whereas the cross-sectional validation analysis sample at baseline comprised 229 patients due to 2 patients not providing any item-level QOL-B RD data. Due to rounding, percentages may total more than 100%.

Abbreviations: BMI, body mass index; FEV_1_, forced expiratory volume in 1 second; QOL-B RD, Quality of Life-Bronchiectasis Respiratory Domain.

Item-level Descriptive Assessments

There were no methodological differences in item-level descriptive assessments for the 9-item QOL-B RD analysis. Although the presence of floor effects was indicated for 4 of the 9 items (44%, **Table S5**), the items’ other response categories were reasonably endorsed by patients, demonstrating the items’ utility. Overall, 27 of the 45 inter-item correlations (60%) met or exceeded r = 0.4 (**Table S6**). There was no distinct pattern in the 13 correlations less than or equal to 0.2. Consequently, results indicate that the QOL-B RD is largely composed of items expected to define a monolithic score, with varying strengths of association and no evidence of redundant items.

Table S5. Item Response Pattern: 9-item QOL-B RD Analysis

| **QOL-B RD Item** | **N (%) of Participants** | | | | |
| --- | --- | --- | --- | --- | --- |
|  | **A Lot** | **A Moderate Amount** | **A Little** | **Not at All** | **Missing** |
| Have you felt congestion in your chest? | 24 (10.43) | 56 (24.35) | 109 (47.39) | 40 (17.39) | 1 (0.43) |
| Have you been coughing during the day? | 33 (14.35) | 68 (29.57) | 113 (49.13) | 15 (6.52)^a^ | 1 (0.43) |
| Have you had to cough up mucus? | 30 (13.04) | 78 (33.91) | 103 (44.78) | 18 (7.82)^a^ | 1 (0.43) |
|  | **Clear** | **Clear-to-Yellow** | **Yellowish-Green** | **Brownish-Dark or Green with Traces of Blood** | **Missing** |
| Has your sputum been mostly…? | 36 (15.65) | 113 (49.13) | 51 (22.17) | 20 (8.70)^a^ | 10 (4.35) |
|  | **Always** | **Often** | **Sometimes** | **Never** | **Missing** |
| Have you had shortness of breath with greater activity, such as housework or yardwork? | 40 (17.39) | 46 (20) | 105 (45.65) | 38 (16.52) | 1 (0.43) |
| Have you had wheezing? | 2 (0.87)^a^ | 16 (6.96)^a^ | 95 (41.30) | 116 (50.43)^b^ | 1 (0.43) |
| Have you had chest pain? | 3 (1.30)^a^ | 14 (6.09)^a^ | 96 (41.74) | 116 (50.43)^b^ | 1 (0.43) |
| Have you had shortness of breath when talking? | 6 (2.61)^a^ | 36 (15.65) | 103 (44.78) | 84 (36.52)^b^ | 1 (0.43) |
| Have you woken up during the night because you were coughing? | 7 (3.04)^a^ | 20 (8.70)^a^ | 110 (47.83) | 92 (40)^b^ | 1 (0.43) |

^a^ Sparseness. ^b^ Floor effect.

Abbreviation: QOL-B RD, Quality of Life-Bronchiectasis Respiratory Domain.

Table S6. Inter-Item Polychoric Correlations at Baseline: 9-item QOL-B RD Analysis

| **QOL-B RD Item** | **Chest Congestion** | **Cough Day** | **Cough Mucus** | **Sputum Color^a^** | **SB Greater Activity** | **Wheeze** | **Chest Pain** | **SB Talking** | **Cough Woken** |
| --- | --- | --- | --- | --- | --- | --- | --- | --- | --- |
| Chest Congestion | 1.00 |  |  |  |  |  |  |  |  |
| Cough Day | 0.49 | 1.00 |  |  |  |  |  |  |  |
| Cough Mucus | 0.45 | 0.47 | 1.00 |  |  |  |  |  |  |
| Sputum Color^a^ | 0.36^a^ | 0.23 | 0.34 | 1.00 |  |  |  |  |  |
| SB Greater Activity | 0.44 | 0.13^b^ | 0.19^b^ | 0.25 | 1.00 |  |  |  |  |
| Wheeze | 0.45 | 0.07^b^ | 0.22 | 0.27 | 0.42 | 1.00 |  |  |  |
| Chest Pain | 0.55 | 0.22 | 0.17^b^ | 0.23 | 0.50 | 0.47 | 1.00 |  |  |
| SB Talking | 0.48 | 0.17^b^ | 0.17^b^ | 0.17^b^ | 0.76 | 0.52 | 0.49 | 1.00 |  |
| Cough Woken | 0.54 | 0.41 | 0.49 | 0.36^a^ | 0.33 | 0.63 | 0.41 | 0.32 | 1.00 |

^a^ Correlation fell below 0.4 but were in rounding of 0.4.

^b^ Correlations fell below ≤ 0.2.

Abbreviations: QOL-B RD, Quality of Life-Bronchiectasis Respiratory Domain; SB, shortness of breath.

Modern Psychometric Methods

The iEFA, IRT, LD, and DIF methodology for the 9-item QOL-B RD analysis were the same as for the 8-item respiratory symptom score analysis with one exception: Bock's nominal likelihood was employed in the iEFA model for item 32 (sputum color) [32-34], while Samejima’s GRM was used for all other items. The nominal model is the latent variable analog of a multinomial logistic regression.

The modern psychometric methods supported the relevance of all items and an essentially unidimensional unit-weighted mean score for the QOL-B RD. Item parceling was explored for cough and dyspnea due to persistent LD; the parceled bifactor model had acceptable model fit, dimensionality, and scoring statistics (RMSEA lower CI limit: 0.024; omega ratio: 0.997) and all LD was eliminated (**Table S7**). IRT predicted item response probabilities (item response functions) indicated strong measurement of respiratory function. During the DIF sweep, item 33 (‘shortness of breath with greater activity’) had potential differential functioning across age groups, but an exploration of DTF showed that age group differences on this item had functionally no effect on QOL-B RD scores. Specifically, the DTF results indicated a very weak effect on scores for the age DIF associated with this item. Plotting the test scoring functions stratified on the age median-split demonstrated that the test scoring functions did not separate and nearly completely overlapped in their 95% confidence bands (**Figure S1**). Because the pre-specified success criteria were met, an essentially unidimensional unit-weighted score appears to be empirically justified for the QOL-B RD within this context of use.

Table S7. Model Fit Comparisons: 9-item QOL-B RD Analysis

|  | RMSEA (90% CI) | TLI | CFI | Omega | Hierarchical Omega | Omega Ratio | H-statistic |
| --- | --- | --- | --- | --- | --- | --- | --- |
| 2-factor MIRT | 0.114  (0.091, 0.139) | 0.873 | 0.912 | 0.800 | - | - | - |
| 2-factor Bifactor | 0.000  (0.000, 0.059) | 1.002 | 1.000 | 0.880 | 0.647 | 0.735 | 0.837 |
| 3-factor MIRT | 0.142  (0.119, 0.166) | 0.804 | 0.864 | 0.776 | - | - | - |
| 3-factor Bifactor | 0.108  (0.078, 0.138) | 0.888 | 0.950 | 0.869 | 0.731 | 0.841 | 0.828 |
| Item parceling analysis | | | | | | | |
| Parceled Bifactor Model | 0.082 (0.024, 0.141) | 0.934 | 0.984 | 0.829 | 0.827 | 0.997 | 0.845 |

Abbreviations: CI, confidence interval; CFI, comparative fit index; MIRT, multidimensional item response theory; QOL-B RD, Quality of Life-Bronchiectasis Respiratory Domain; RMSEA, root mean squared error of approximation; TLI, Tucker-Lewis index.

Figure S1. Modern Psychometric Methods: 9-item QOL-B RD Test Scoring Function Stratified on Age Median-Split

Abbreviation: QOL-B RD, Quality of Life-Bronchiectasis Respiratory Domain.

Reliability and Validity

Classical test theory statistics of reliability and validity, as measured by internal consistency (**Table S8**), test-retest reliability (ICC[2,1]: 0.69), and construct validity (**Table S9, Table S10**), demonstrated that the 9-item QOL-B RD score appears to be psychometrically robust in newly diagnosed MAC lung disease, whereby all pre-specified success criteria were met.

Table S8. Reliability: Internal Consistency Estimates at Baseline: 9-item QOL-B RD Analysis

| **QOL-B RD Items** | **McDonald’s omega (95% CI)** | **Cronbach’s alpha (95% CI)** | **Item-Total Polyserial Correlation** | **Cronbach’s alpha  If Item Is Dropped** |
| --- | --- | --- | --- | --- |
| Domain score | 0.80 (0.76, 0.84) | 0.79 (0.75, 0.83) |  |  |
| Chest Congestion |  |  | 0.83 | 0.75 |
| Cough Day |  |  | 0.58 | 0.79 |
| Cough Mucus |  |  | 0.62 | 0.78 |
| Sputum Color |  |  | 0.57 | 0.79 |
| SB Greater Activity |  |  | 0.73 | 0.77 |
| Wheeze |  |  | 0.64 | 0.78 |
| Chest Pain |  |  | 0.70 | 0.77 |
| SB Talking |  |  | 0.72 | 0.77 |
| Cough Woken |  |  | 0.73 | 0.77 |

Abbreviations: CI, confidence interval; QOL-B RD, Quality of Life-Bronchiectasis Respiratory Domain; SB, shortness of breath.

Table S9. Concurrent Validity at Baseline: 9-item QOL-B RD Analysis

|  | **EXACT^a^** | **E-RS^a^** | **FACIT-Fatigue^b^** | **SGRQ^a^** |
| --- | --- | --- | --- | --- |
| QOL-B RD score^b^ | -0.70 | -0.70 | 0.49 | -0.71 |

^a^EXACT, E-RS, and SGRQ: Higher score = higher severity, lower score = lower severity.

^b^FACIT-Fatigue and QOL-B RD: Higher score = lower severity, lower score = higher severity.

Abbreviations: CI, confidence interval; E-RS, EXACT Respiratory Symptoms; EXACT, Exacerbations of Chronic Pulmonary Disease Tool; FACIT-Fatigue, Functional Assessment of Chronic Illness Therapy Fatigue Scale; QOL-B RD, Quality of Life-Bronchiectasis Respiratory Domain; SGRQ, St. George Respiratory Questionnaire.

Table S10. Known-Groups Validity at Baseline: 9-item QOL-B RD Analysis

| **PGI-S Respiratory Reference Category** | **PGI-S Respiratory Effect Category** | **N** | **LSM Estimate (95% CI)** | **LSM Contrast** | ***P*-value** | **Semi-Partial Omega Squared (95% CI)** |
| --- | --- | --- | --- | --- | --- | --- |
| Not at all |  | 24 | 80.42 (75.75, 85.09) |  |  |  |
|  | Mildly | 100 | 71.18 (68.90, 73.45) | 9.24 | < 0.01 | 0.13 (0.06, 0.21) |
|  | Moderately | 70 | 59.56 (56.82, 62.29) | 20.86 | < 0.001 | 0.36 (0.27, 0.45) |
|  | Very | 33 | 40.61 (36.63, 44.60) | 39.81 | < 0.001 | 0.45 (0.36, 0.53) |
|  | Extremely | 2 | 62.96 (61.25, 79.14) | 17.46 | 0.25 | 0.20 (0.12, 0.29) |

Abbreviations: CI, confidence interval; LSM, least squares means; PGI-S, Patient Global Impression of Severity; QOL-B RD, Quality of Life Questionnaire–Bronchiectasis Respiratory Domain.

Meaningful Change

Prior to the MWPC analysis, Pearson correlations between the 9-item QOL-B RD change scores and PGI-S Respiratory change scores at end of study (1-month off treatment) were calculated. A correlation of -0.52 indicated the PGI-S Respiratory was a suitable anchor for use in these analyses. Findings from anchor-based analyses indicated that a 14.81-point increase (95% CI, 8.82-18.52 points) in the 9-item QOL-B RD score from baseline is a clinically meaningful within-patient improvement for an adult population with newly diagnosed MAC lung disease (**Table S11**). The eCDF curves showed clear and consistent separation between the improved and maintained anchor groups across all QOL-B RD score changes, including the 14.81 MWPC threshold (**Figure S2**).

Table S11. Anchor-based Mean and Median Change from Baseline in 9-item QOL-B RD Score at End of Study (N=99 ARISE Patients)^a^

| **Thresholds by Anchor Group** | | | |
| --- | --- | --- | --- |
| **Anchor Group** | **Mean (95% CI)** | **Median (95% CI)** | **N (%)** |
| Improved, 2+ categories | 24.40 (13.70, 35.09) | 25.93 (11.11, 29.63) | 12 (12.1) |
| Improved, 1 category | 14.77 (10.00, 19.55) | 14.81 (8.82, 18.52) | 35 (35.4) |
| Maintained | 6.49 (2.57, 10.41) | 3.70 (0.00, 11.11) | 41 (41.4) |
| Deteriorated, 1 category | 4.63 (-4.09, 13.35) | 3.70 (-7.41, 14.81) | 8 (8.1) |
| Deteriorated, 2+ categories | -28.40 (NA)^b^ | -37.04 (NA)^b^ | 3 (3.0) |

^a^End of study: 1 month off treatment (month 7).

^b^Sample size did not permit calculation of confidence intervals.

Abbreviations: CI, confidence interval; NA, not applicable; QOL-B RD, Quality of Life-Bronchiectasis Respiratory Domain.

Figure S2. eCDF of the 9-Item QOL-B RD Change Score from Baseline to End of Study by PGI-S Respiratory Anchor Group (N=99 ARISE Patients)^a^

^a^End of study: 1 month off treatment (month 7).

Abbreviations: eCDF, empirical cumulative distribution function; PGI-S, Patient Global Impression of Severity; QOL-B RD, Quality of Life-Bronchiectasis Respiratory Domain.

Discussion

Anchor-based MWPC results supported a 14.81-point median change from baseline as a potential threshold for clinically meaningful within-patient improvement for the 9-item QOL-B RD.

Overall, the 9-item QOL-B RD, a well-established PRO instrument with well-understood measurement properties, appears to be a psychometrically robust PRO for use in adult patients with a new or recurrent diagnosis of MAC lung disease and appears sensitive to measure a meaningful change in respiratory symptoms.

Appendix A. Essential Dimensionality Review

The subfield of psychometrics dedicated to essential dimensionality is concerned with a single question: to what extent can a unidimensional framework (score or model) approximate a multidimensional test without inducing unacceptably high measurement error. This question arose in the late 1970s when educational researchers recognized that the software used to model and score tests was restricted to the unidimensional framework while the tests were reasonably assumed to be multidimensional. The field conjectured that so long as a dominant dimension accounted for the majority of variance in the test then the unidimensional approximation would not induce measurement error sufficient to compromise the test. This conjecture was directly related to the idea of general intelligence (g) debated in the early 20^th^ century literature and formally tested by Holzinger & Swineford (1937) [35] who defined the bifactor structure for g and whose work would be later operationalized by Gibbons and Hedeker (1992) [36] in their seminal dimension reduction algorithm. Reckase (1979) [37] demonstrated preliminary validity for this theory with his pioneering simulation. Intermittent contributions to the literature occurred over the 1980s, with the technical mathematical definition being given by Stout (1990) [18], who, in addition to providing the mathematical definition for essential unidimensionality, provided multiple theorems defining conditions identifying the presence of essential unidimensionality in real data.

(Essential) dimensionality is defined as the minimum number of dimensions required to minimize (essential independence) or eliminate (conditional independence) local dependence. When a dominant general domain is present and the corresponding bifactor model minimizes or eliminates local dependence, then the test may be essentially unidimensional [18, 37-39]. Achieving criteria satisfying essential unidimensionality supports the determination, according to Stout (1990) [18], that “minor or idiosyncratic dimensions should be ignored in assessing test dimensionality from the applications viewpoint”.

This philosophical approach was extended to a series of effect-size statistics designed to empirically test and quantify the measurement error associated with treating a test or score as essentially unidimensional [19, 40-42]. This literature and the resulting statistics, as described in Rodriguez, Reise, & Haviland (2016) [20], form the basis of our dimensionality assessment. These statistics, derived from the contemporary literature, are described next [19, 41, 43, 44].

A determination of essential unidimensionality would demonstrate that while, for example, *COUGH* and *DYSPNEA* subdomains may exist, they may weakly account for variance in a unit-weighted score relative to the influence of the overarching construct of *MAC lung infection* *symptoms* (note the use of capitalization and italics to denote domain names as opposed to item names).

Thus, essential unidimensionality does not require an omega ratio of 1, as an omega ratio of 1 is not evidence of essential unidimensionality but rather absolute unidimensionality, and virtually nothing is truly absolutely unidimensional but most things are essentially unidimensional.

As for the use of omega ratios for evaluating essential dimensionality, what was written and what was done is as follows:

McDonald’s $\omega$ and related statistics are useful in determining the essential unidimensionality of a unit-weighted total score [19]. Unit-weighted total scores can be unidimensional to greater and lesser degrees, even for a strictly unidimensional test, depending on the strength of minor factors influencing the responses [38]. McDonald’s $\omega$ represents the proportion of variance in a unit-weighted total score attributable to all factors. McDonald’s $\omega_{H}$, where H stands for hierarchical, represents the proportion of variance in a unit-weighted total score that can be attributed to the general factor in a bifactor model. McDonald’s $\omega_{HS}$, where HS stands for hierarchical subscale, represents the proportion of variance in a unit-weighted total score that can be attributed to the specific factors in a bifactor model, with one $\omega_{HS}$ estimated for each specific factor. When $\omega_{H}$ exceeds 0.8 the general factor explains 80% of the variance in the unit-weighted total score. When the ratio of $\omega_{H}$ to $\omega$, ${\omega_{H}}/\omega$, exceeds 0.8 then out of all sources of variance in the unit-weighted total score the general factor is dominant and the specific factor(s) only account for 20% of the variance in the unit-weighted total score. In such a circumstance the unit-weighted total score is essentially unidimensional.

Our estimated omega ratio (0.773) is within rounding error of 0.8. For this reason, we are confident that treating the 8-item respiratory symptom score as essentially unidimensional is reasonable and compatible with the developer’s instructions and the extant literature describing all other applications that have treated the QOL-B RD as a unidimensional score. Omission of a single item in the presence of this essential dimensionality evidence does not lead us to the conclusion that the score is multidimensional or requires empirical weighting.

References

1.	NCT04677543: Validation of patient reported outcome measures in participants with nontuberculous mycobacterial lung infection caused by Mycobacterium avium complex (ARISE): [cited 2024 May 20]. Available from: https://clinicaltrials.gov/study/NCT04677543

2.	NCT04677569: Study to evaluate ALIS (amikacin liposome inhalation suspension) in participants with nontuberculous mycobacterial lung infection caused by Mycobacterium avium complex (ENCORE): [cited 2024 May 20]. Available from: https://www.clinicaltrials.gov/study/NCT04677569

3.	Mange KC, Serrano D, Hassan M, et al. Psychometric validation of the PROMIS Fatigue-Short Form 7a in adults with newly diagnosed or recurrent Mycobacterium avium complex (MAC) lung disease: the ARISE and ENCORE studies. J Patient Rep Outcomes. 2025;9(1):119.

4.	Quittner AL, O'Donnell AE, Salathe MA, et al. Quality of Life Questionnaire-Bronchiectasis: final psychometric analyses and determination of minimal important difference scores. Thorax. 2015;70(1):12-20.

5.	McCarrier KP, Hassan M, Yuen DW, et al. Content validation of the QOL-B-RD and PROMIS-F SF-7a to measure respiratory and fatigue symptoms of MAC lung disease. Adv Ther. 2025;42(2):935-976.

6.	US Food and Drug Administration. Patient-focused drug development: incorporating clinical outcome assessments into endpoints for regulatory decision-making. Draft guidance for industry, Food and Drug Administration staff, and other stakeholders: 2023 [cited 2024 June 25]. Available from: https://www.fda.gov/media/166830/download

7.	R Core Team. R: a language and environment for statistical computing. Vienna: R Foundation for Statistical Computing; 2023. Available from: https://www.R-project.org

8.	Revelle W. Psych: procedures for psychological, psychometric, and personality research. R package version 2.3.6. Evanston, Illinois: Northwestern University. Available from: https://CRAN.R-project.org/package=psych

9.	Chalmers RP. mirt: a multidimensional item response theory package for the R environment. J Stat Softw. 2012;48(6):1-29.

10.	Fox J. polycor: Polychoric and Polyserial Correlations. R package. 2022;Version 0.8.1: 2022. Available from: https://cran.r-project.org/web/packages/polycor/polycor.pdf

11.	McGraw KO, Wong SP. Forming inferences about some intraclass correlation coefficients. Psychol Methods. 1996;1(1):30-46.

12.	Gamer M, Lemon J, Singh I. irr: Various Coefficients of Interrater Reliability and Agreement. Version 0.84.1: 2010. Available from: https://cran.r-project.org/web/packages/irr/irr.pdf

13.	Lenth R. emmeans: Estimated Marginal Means, aka Least-Squares Means. R package 2023; Version 1.8.7. Available from: https://cran.r-project.org/web/packages/emmeans/emmeans.pdf

14.	Ben-Shachar MS, Lüdecke D, Makowski D. effectsize: Estimation of effect size indices and standardized parameters. J Open Source Softw. 2020;5(56):2815.

15.	Samejima F. Estimation of latent ability using a response pattern of graded scores. Psychometrika Monogr Supplement. 1969;34(4, Pt. 2):100.

16.	MacCallum RC, Browne MW, Sugawara HM. Power analysis and determination of sample size for covariance structure modeling. Psychol Methods. 1996;1:130-149.

17.	Hu LT, Bentler PM. Cutoff criteria for fit indexes in covariance structure analysis: Conventional criteria versus new alternatives. Struct Eq Model Multidiscipl J. 1999;6(1):1-55.

18.	Stout WF. A new item response theory modeling approach with applications to unidimensionality assessment and ability estimation. Psychometrika. 1990;55(2):293-325.

19.	McDonald RP. Test theory: a unified treatment. Mahwah, NJ: Lawrence Erlbaum Associates; 1999.

20.	Rodriguez A, Reise SP, Haviland MG. Evaluating bifactor models: Calculating and interpreting statistical indices. Psychol Methods. 2016;21(2):137-150.

21.	Chen W-H, Thissen D. Local dependence indexes for item pairs using item response theory. J Educ Behav Stat. 1997;22(3):265-289.

22.	Woods CM, Cai L, Wang M. The Langer-improved Wald test for DIF testing with multiple groups: evaluation and comparison to two-group IRT. Educ Psychol Meas. 2013;73(3):532-547.

23.	Wald A. Tests of statistical hypotheses concerning several parameters when the number of observations is large. Trans Am Math Soc. 1943;54(3):426-482.

24.	Clauser BE, Mazor KM. Using statistical procedures to identify differentially functioning test items. Educat Measure. 1998;17(1):31-44.

25.	Scott NW, Fayers PM, Aaronson NK, et al. A simulation study provided sample size guidance for differential item functioning (DIF) studies using short scales. J Clin Epidemiol. 2009;62(3):288-295.

26.	Yu L, Moses T, Puhan G, et al. DIF detection with small samples: applying smoothing techniques to frequency distributions in the Mantel-Haenszel procedure. ETS Res Report Ser. 2008;2008(2):i-33.

27.	Zwick R. A review of ETS differential item functioning assessment procedures: flagging rules, minimum sample size requirements, and criterion refinement. ETS Res Report Ser. 2012;2012(1):i-30.

28.	Hansen M, Cai L, Stuckey BD, et al. Methodology for developing and evaluating the PROMIS® smoking item banks. Nicotine Tob Res. 2013;16(Suppl 3):S175-S189.

29.	Cohen J. Statistical power analysis for the behavioral sciences. 2nd ed.: Lawrence Erlbaum Associates; 1988.

30.	Chalmers RP, Counsell A, Flora DB. It might not make a big DIF: improved differential test functioning statistics that account for sampling variability. Educ Psychol Meas. 2016;76(1):114-140.

31.	Meade AW. A taxonomy of effect size measures for the differential functioning of items and scales. J Appl Psychol. 2010;95(4):728-743.

32.	Bock RD. Estimating item parameters and latent ability when responses are scored in two or more nominal categories. Psychometrika. 1972;37(1):29-51.

33.	Thissen D, Cai L, Bock RD. The nominal categories item response model. In: Nering ML, Ostini R, editors. Handbook of polytomous item response theory models. New York: Routledge; 2010. p. 43-75.

34.	Bock RD. The nominal categories model. In: van der Linden W, Hambleton RK, editors. Handbook of modern item response theory. New York: Springer; 1997. p. 33-50.

35.	Holzinger KJ, Swineford F. The bi-factor method. Psychometrika. 1937;2(1):41-54.

36.	Gibbons RD, Hedeker DR. Full-information item bi-factor analysis. Psychometrika. 1992;57(3):423-436.

37.	Reckase MD. Unifactor latent trait models applied to multifactor tests: results and implications. J Educ Stat. 1979;4(3):207-230.

38.	Nandakumar R. Traditional dimensionality versus essential dimensionality. J Educ Meas. 1991;28(2).

39.	Nandakumar R, Yu F, Li H-H, Stout W. Assessing unidimensionality of polytomous data. Appl Psychol Meas. 1998;22(2):99-115.

40.	Grice JW. Computing and evaluating factor scores. Psychol Methods. 2001;6(4):430.

41.	Hancock GR. Effect size, power, and sample size determination for structured means modeling and mimic approaches to between-groups hypothesis testing of means on a single latent construct. Psychometrika. 2001;66(3):373-388.

42.	Hancock G, Mueller R. Rethinking construct reliability within latent variable systems. In: Robert Cudeck KGJ, Dag Sörbom, Stephen Du Toit, ed. Structural equation modeling: present and future: a Festschrift in honor of Karl Jöreskog. Scientific Software International; 2001:195-216.

43.	Ten Berge J, Sočan G. The greatest lower bound to the reliability of a test and the hypothesis of unidimensionality. Psychometrika. 2004;69(4):613-625.

44.	Raykov T, Pohl S. Essential unidimensionality examination for multicomponent scales:an interrelationship decomposition approach. Educ Psychol Meas. 2013;73(4):581-600.
